# Short‐term impacts of COVID‐19 on food security and nutrition in rural Guatemala: Phone‐based farm household survey evidence

**DOI:** 10.1111/agec.12629

**Published:** 2021-05-02

**Authors:** Francisco Ceballos, Manuel A. Hernandez, Cynthia Paz

**Affiliations:** ^1^ Markets, Trade and Institutions Division International Food Policy Research Institute Washington District of Columbia USA

**Keywords:** agricultural households, COVID‐19, dietary diversity, food security, income

## Abstract

This article examines the short‐term effects of the COVID‐19 lockdown on food security and nutrition in rural Guatemala. We rely on a comprehensive panel dataset of 1,824 small agricultural households collected over two survey rounds, on November–December 2019 and May–June 2020. We place special emphasis on changes in agricultural and nonagricultural income sources, including remittances, and changes in dietary diversity, including consumption of animal source foods (ASF) and fruits and vegetables (F&V). We find that COVID‐19 affected the incomes, food security, and dietary patterns of households, with a decrease in ASF diversity and an increase in F&V diversity, and an overall net decrease in dietary diversity across all food groups. Dietary diversity among women in reproductive age, however, remained unchanged, and increased among children under 2 years old. Interestingly, households with relatively higher incomes appear to have reduced their dietary diversity to a larger extent than lower income ones, as well as households located in communities with more severe access restrictions. The focus of the study in a region with a high prevalence of poverty and chronic malnutrition provides an important perspective into the consequences of the lockdown in complex rural contexts with vulnerable populations and contributes to inform eventual recovery measures.

## 1 INTRODUCTION

The rapid spread of the novel coronavirus (COVID‐19) outbreak and the drastic measures incurred to curtail it during the first half of 2020 represent a major global economic shock. Projections by The World Bank indicate a global gross domestic product (GDP) contraction above 5%—the sharpest recession since 1960— pushing between 71 and 100 million people into extreme poverty (World Bank, 2020). Such scenarios entail serious consequences in terms of food security and nutrition, particularly for vulnerable smallholders in developing countries. The World Food Programme (WFP) estimates that an additional 130 million households will become food insecure in the course of 2020, almost doubling their 2019 baseline numbers (WFP, 2020). Latin American and the Caribbean economies are not exempt from these global downturns, with an estimated GDP contraction of 7.2% and 9 million additional food‐insecure households (a 50% increase).

We focus on the case of Guatemala, where the government imposed strict measures in reaction to COVID‐19. On March 11, 2020, the country introduced travel restrictions following the World Health Organization's (WHO) declaration of COVID‐19 as a global pandemic and subsequently instituted a ban on any public events and agglomerations of more than 100 people on March 15 (2 days after the first case was reported) and a nationwide lockdown on March 21. The latter involved measures such as a temporary halt of activities in the private and public sectors, suspension of public transportation, and restricted mobility of residents, with a strict curfew from 6 pm to 5 am every day. To put these measures in perspective, the government response stringency index for Guatemala (as estimated by Roser et al., 2020) was of 96.3 out of 100 between April 18 and July 26, above that of most neighboring countries (except Honduras) and other North and South American economies.^1^

Such strict social and economic measures are expected to have important effects on Guatemala's economy. Recent reports for Guatemala indicate a 10.2% decrease in the economic activity and a 20.2% decrease (year‐over‐year) in remittances as of April 2020.^2^ In terms of the rural economy, while agricultural activities were exempted from the government restrictions, agricultural and food supply chains have still been affected by limitations to the mobility of people and goods and are expected to continue suffering from contractions in both the supply and demand for agricultural products, disruptions in trade and logistics, and labor shortages due to widespread lockdown measures (Food and Agriculture Organization & Economic Commission for Latin America and the Caribbean, 2020a and 2020b). Moreover, the country was recently categorized as of medium‐high and high risk in terms of its exposure to COVID‐19 impacts on, respectively, the supply and demand for food products (Food and Agriculture Organization & Community of Latin American and Caribbean States, 2020). Ultimately, the effects of the pandemic are yet unclear and will depend on several crucial factors, including the ongoing interaction between supply and demand effects, how market actors and stakeholders anticipate and respond to these, and the success in containing the virus.

This study aims to partially fill this gap. We assess the short‐term effects of COVID‐19 on food security and nutrition of vulnerable populations in rural Guatemala. The study builds on a recent survey conducted in November–December 2019 (before the start of the pandemic) to agricultural households owning a cellphone, located across 86 communities in the departments of Huehuetenango, Quiche, and San Marcos. During May–June 2020, a brief follow‐up phone survey was administered among these households, allowing us to analyze the short‐term effects of the COVID‐19 restrictions on several relevant food security and dietary diversity outcomes. In particular, the analyses focus on variations in income sources and food consumption patterns before and after the start of the pandemic, while accounting for a wide set of household characteristics, together with location and features of the food environment. Special emphasis is placed on changes in agricultural and nonagricultural incomes, including remittances, and changes in dietary diversity, including dietary diversity among women in reproductive age and children under 2 years old and consumption (access) of animal source foods (ASF) and fruits and vegetables (F&V).

We find that COVID‐19 appears to have affected the incomes, food security, and dietary patterns of rural households in the region. Interviewed households report a decrease in their agricultural and nonagricultural income and in their income from remittances. Similarly, most households report reduced food availability in local markets and higher prices, which combined with lower earnings seem to have contributed to a higher occurrence of experiences of food insecurity since the start of the COVID‐19 lockdown. Moreover, households report an overall decrease in diversity across all food groups, with a decrease in ASF diversity and an increase in F&V diversity. Dietary diversity among women, however, remained unchanged, and increased among young children. The results also point to a larger reduction in dietary diversity among households with relatively higher incomes, and among households located in communities with more strict access restrictions due to the pandemic.

Our study relates to several strands of the broader literature on household resilience and nutritional effects from shocks. Reductions in income or increases in food prices have been found to lead to substitution across food categories (Choudhury et al., 2019; Hoang, 2018; Jensen & Miller, 2008) and a decrease in the quality of diets (Brinkman et al., 2010). With poor households spending a large share of their incomes on basic foods (Ivanic & Martin, 2008), this can lead to increased food insecurity and periods of malnutrition with both short‐term and long‐term health, productivity, employment, and income consequences (Akter & Basher, 2014; Niles & Salerno, 2018; Shah & Steinberg, 2017; Smith & Frankenberger, 2018; Smith & Glauber, 2019). Closer to our study, Abate et al. (2020) find that the COVID‐19 crisis negatively affected urban household incomes and food security in Ethiopia (Addis Ababa), with disproportionate effects on the least wealthy households.

The remainder of the article is organized as follows. Section 2 describes the data sources and the procedure around data collection, together with a description of the sampled households and the key outcome measures. Section 3 discusses the methods employed for the empirical analyses and potential issues that could affect our results. Section 4 presents the results discussing changes in income, food security, and dietary diversity indicators, including identifying specific household patterns and potential factors associated with these changes. Section 5 offers some concluding remarks and policy recommendations.

## 2 DATA

**FIGURE 1:**
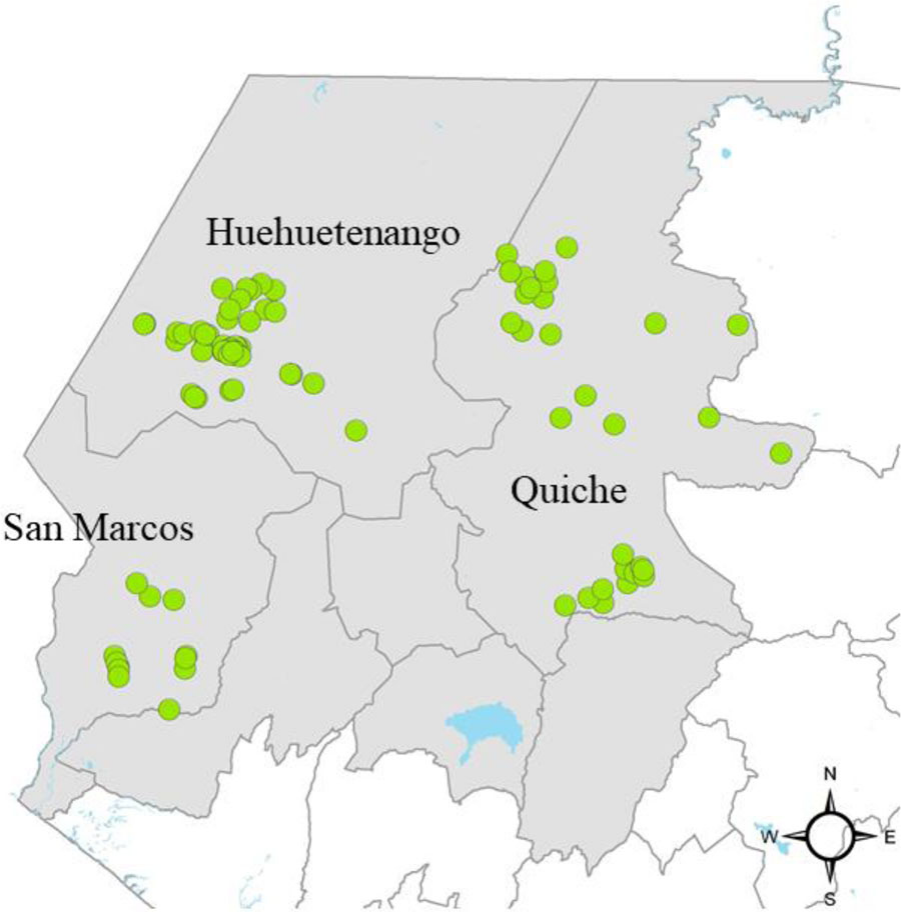
Sampled communities in the panel survey [Color figure can be viewed at wileyonlinelibrary.com] *Note*: This figure maps the average georeferenced point (green circle) of all interviewed households within each sampled community in the panel survey

The sample frame for the study includes households involved in commercial smallholder farming that were interviewed towards the end of 2019, as part of a baseline survey for a broader impact evaluation of a value chain development program for coffee and horticultural crops in the Western Highlands of Guatemala.^3^ This region is arguably the most vulnerable area in the country and a focal point for aid and development programs.^4^ The sample frame includes 2,142 households located across the departments of Huehuetenango, Quiche, and San Marcos, who provided a cell phone number. A subset of these households is a randomly selected, representative sample of the total population of program beneficiaries, with the remaining households belonging to nonbeneficiary neighboring communities with similar characteristics to those within the program's reach.^5^ Figure 1 maps the 86 communities included in the sample.

Our sample includes a large proportion of households that produce coffee as one of their main crops as well as some producers of cardamom and horticultural crops, combined with staple crops such as maize and black beans. As such, it is not necessarily representative of all the rural population in the Western Highlands. The sample poverty rate (77.2%) and chronic malnutrition rate for children under 2 years old (52.1%) are still within the department‐level rates, ranging between 65.5–81% and 45.4–63.1%, respectively ( Instituto Nacional de Estadistica, 2015; Ministerio de Salud Publica y Asistencia Social, Instituto Nacional de Estadistica, & ICF International, 2017). We further discuss below the comparability of the study sample with the average rural household in the region and the potential generalization of our findings.^6^

We rely on a panel dataset comprising the baseline and a follow‐up survey. The baseline survey, conducted in November–December 2019, was administered through in‐person interviews and covered an extensive range of topics, including demographic characteristics, agricultural production, assets and income sources, household expenditure, participation in social programs, food security, dietary diversity, and anthropometry measurement for women and children. The follow‐up survey, implemented in May–June 2020, consisted of a short survey specifically designed to inquire about the potential economic and nutritional effects of the lockdown and other COVID‐19‐related measures. The survey included modules on qualitative changes in income sources (including remittances) after the lockdown, coping mechanisms, food security indicators, and dietary diversity.

The follow‐up survey was conducted exclusively by phone, relying on the same (female) local enumerators and phone numbers collected during the first round. To encourage participation and to appreciate households for their time in responding, we provided each interviewed household with a small airtime top‐up (equivalent to 1.33 U.S. dollars). We also relied on the community leaders to contact households that did not answer repeated phone calls, as some of them had lost or changed their phone numbers, a common occurrence in rural Guatemala.^7^ The final study sample is a panel of 1,824 households who were located and interviewed in both survey rounds.^8^

Table 1 provides summary statistics of a wide set of socioeconomic characteristics (measured at baseline) of our working sample, including characteristics of the household head (gender, age, education, main language spoken); general household characteristics (household size, whether it is a beneficiary of a social school program, dwelling materials, per capita expenditure, and access to services); asset ownership; agricultural activities (main crops cultivated, livestock ownership, land size); if a household member is an internal migrant (worker); whether the household is beneficiary of the Value Chains Project; and location. As shown, most households in our sample have a male household head with no or incomplete primary education, and more than two thirds speak a language other than Spanish as their primary language. In addition, the average household has almost six members and has a TV or radio. The dwelling typically has finished floors, unfinished ceiling, is connected to electricity and to a water system, but lacks connection to a drainage network. These socioeconomic characteristics generally resemble the representative household from the three departments covered in the study, but are somewhat better off than the average rural household in the area.^9^ The average agricultural land size is less than 1 hectare, four out of every five households produce coffee as one of their main crops, while one out of every two households produce corn as one of their main crops. The daily average per capita expenditure is 11.8 quetzales (equivalent to 1.57 U.S. dollars) and practically all households (99%) reported receiving income from agricultural activities in 2019, while 56% reported income from nonagricultural activities, and 25% received remittances.

**TABLE 1:** Household characteristics at baseline (November–December 2019)

Variable	Mean	Standard Deviation	Minimum	Maximum
If household head is male	0.833	0.373	0.000	1.000
Household head age	48.049	14.074	16.000	94.000
If household head has no education	0.351	0.477	0.000	1.000
If household head did not complete elementary education	0.318	0.466	0.000	1.000
If household head completed elementary education or above	0.331	0.471	0.000	1.000
If household head main language spoken is Spanish	0.320	0.467	0.000	1.000
Household size	5.764	2.681	1.000	22.000
If household is beneficiary of social school program	0.273	0.446	0.000	1.000
If dwelling has finished walls	0.479	0.500	0.000	1.000
If dwelling has finished ceiling	0.143	0.350	0.000	1.000
If dwelling has finished floor	0.561	0.496	0.000	1.000
If dwelling is connected to electricity	0.850	0.357	0.000	1.000
If dwelling is connected to water system	0.849	0.358	0.000	1.000
If dwelling is connected to drainage network	0.298	0.458	0.000	1.000
If cooking fuel of household is electricity or gas	0.034	0.181	0.000	1.000
Daily per capita expenditure of household (in Quetzales)	11.841	13.422	0.167	108.972
If household owns TV or radio	0.741	0.438	0.000	1.000
If household owns a vehicle	0.240	0.427	0.000	1.000
If household owns livestock	0.572	0.495	0.000	1.000
Agricultural land size of household (in hectares)	0.878	1.384	0.003	21.841
If agricultural land has irrigation system	0.142	0.349	0.000	1.000
If coffee among one of main crops produced	0.798	0.401	0.000	1.000
If cardamom among one of main crops produced	0.116	0.320	0.000	1.000
If corn among one of main crops produced	0.459	0.498	0.000	1.000
If beans among one of main crops produced	0.168	0.374	0.000	1.000
If internal migrant in household last 3 years	0.041	0.197	0.000	1.000
If household is beneficiary of Value Chains Project	0.442	0.497	0.000	1.000
If received income from agricultural activities	0.993	0.081	0.000	1.000
If received income from nonagricultural activities	0.563	0.496	0.000	1.000
If received remittances	0.253	0.435	0.000	1.000
If household located in Huehuetenango	0.563	0.496	0.000	1.000
If household located in Quiche	0.291	0.454	0.000	1.000
If household located in San Marcos	0.146	0.354	0.000	1.000
Observations				1,824

*Note*: This table shows summary statistics for different household characteristics collected during the baseline survey in November–December 2019.

### 2.1 Outcomes of interest

We analyze changes in a series of key outcomes related to the potential effects that the lockdown and other COVID‐19‐related measures (hereafter collectively referred to as “lockdown”) could have had on households’ income sources, food security, and dietary diversity. We construct a wide set of measures to assess the effects of the lockdown on the various outcomes of interest.

First, during the follow‐up survey (May–June 2020), we elicited qualitative self‐reported changes in household incomes, and on their perceptions regarding food availability and food prices in their community, relative to the baseline survey (November–December 2019) for income and relative to the period before the lockdown for food availability and prices.^10^ In terms of income, we inquired about changes to their income from agricultural and nonagricultural activities and their income from remittances. These three income sources represent altogether 94% of the total income received by a household in 2019, as reported in the baseline survey (59% from agricultural activities, 25% from nonagricultural activities, and 10% from remittances).^11^ Due to difficulties in accurately collecting complex (quantitative) measurements over the phone, these indicators are of a qualitative nature and reflect household's perceptions around whether each source of income increased a lot or a little, remained the same, or decreased a lot or a little after the lockdown measures went into effect.^12^ In terms of food availability, we asked households if they had noticed an increase, no change, or a decrease in food availability at their local market. A similar question was asked about the prices for five general categories of foods: grains and cereals, roots, and tubers, F&V, meats, and dairy products.

Second, we compare household answers to three food insecurity questions asked both during the baseline and follow‐up survey. The three food insecurity questions are a subset of the eight items in the Food Insecurity Experience Scale proposed by Ballard et al. (2013) and were selected to capture mild, moderate, and severe experiences of food insecurity. In particular, we asked whether the interviewed individual had experienced a situation since the lockdown where they consumed only a few kinds of foods (mild food insecurity), ate less than they thought they should (moderate food insecurity), and did not eat despite feeling hungry (severe food insecurity) because of a lack of money or other resources.

Third, we focus on dietary diversity outcomes, for which we compare five indicators measured both at baseline and follow‐up. The dietary diversity measures include the household dietary diversity score (HDDS), the women dietary diversity score (WDDS), and a dietary diversity score (DDS) for children between 6 and 23 months of age, widely used in the nutrition literature as a proxy for diet quality and nutrient adequacy (Food and Agriculture Organization, 2010; Ruel, 2003; WHO, 2008).^13^ These three indicators capture the number of food groups consumed over the previous 24 h by, respectively, a reference household member (the interviewed individual), a woman of reproductive age (15–49 years old), and a child 6–23 months old in the household.^14^^,^ 15 The HDDS comprises 12 food groups, while the WDDS comprises nine food groups and the children DDS seven groups.^16^ The dietary diversity indicator for women (WDDS) puts special emphasis on diversity in micronutrient intake, while the indicator for children (DDS) assesses the consumption of basic foods (cereals, grains, roots) plus F&V, animal protein, and dairy products.

Similarly, we analyze baseline and follow‐up diversity in consumption of ASF and F&V, given their importance for achieving a balanced diet (Murphy & Allen, 2003). For these two indicators, we consider the number of food groups consumed by the reference household member, the woman, or the child (over the previous 24 h) among, respectively, seven and five groups of animal‐source foods and F&V.^17^

The focus on dietary diversity indicators is twofold. First, they are measured using a simple count of food groups and are thus easy to collect over a phone survey. Second, although the dietary consumption habits of poor, rural households in the area of study generally consist of a limited amount of foods (with a high presence of cereals and pulses, combined with some vegetables) and do not vary substantially over the year, assessing variations in the number of different food groups consumed (rather than specific quantities) reduces potential biases that may arise from comparing food consumption patterns at different seasons of the year. We return to this discussion in the next section.

## 3 METHODOLOGY

The statistical analyses performed in the study are divided into three sections. We describe below the different empirical methodologies followed in each section and the implications of relying on before–after comparisons in the potential presence of seasonality effects.

Subsection 4.1 shows simple before–after comparisons for the various outcome variables of interest. As indicated above, in the case of income, food availability, and food prices, such comparisons are self‐reported qualitative assessments elicited during the follow‐up survey, relative to the period before the lockdown (baseline period in the case of income). In the case of the food insecurity and dietary diversity measures, we present direct comparisons between the rates or averages collected at the baseline and follow‐up surveys across our study sample.

Subsection 4.2 explores whether household‐level reported variations in income and DDS are associated with various household characteristics. We estimate the following linear probability model via ordinary least squares:^18^

(1) $$Y_{ij}=\alpha +X_{ij\;}\beta +c_{j\;}+u_{ij},$$

where the dependent variable $Y_{ij}$ is an indicator variable capturing whether household *i* in community *j* reported a decrease in income or dietary diversity; $X_{ij}$ is a vector of household characteristics measured at baseline; $c_{j}$ is a location fixed effects component (i.e., a community indicator where the household is located) that controls for community‐level heterogeneity that could be correlated with the observed outcomes (e.g., rural development, social and cultural context, accessibility); and $u_{ij}$ is an idiosyncratic error term. The standard errors are clustered at the community level for likely within‐community correlations in the reported outcomes.

In the case of income, our dependent variable is an indicator variable equal to one if the household reported either a large or small decrease in each income source since December 2019, and zero otherwise.^19^ We combine both large and small decreases as these are self‐reported and could be subject to a (perception) negativity bias due to the pandemic crisis (Baumeister et al., 2001; Godlonton et al., 2018); in particular, the psychology literature argues that people have a strong tendency to overstate negative events.^20^ In the case of dietary diversity outcomes, the dependent variable is an indicator variable equal to one if a given DDS decreased between baseline and follow‐up, and zero otherwise.

For the household characteristics, we rely on a wide set of variables including age, gender, and education of the household head, household size, dwelling characteristics and access to services, terciles of per capita expenditure, asset ownership, agricultural variables such as landholding size and main crops cultivated, and other relevant variables such as internal migration and participation in social programs.^21^ The vector of estimated parameters β in Equation (1) capture the partial correlations between the set of household characteristics and the likelihood of reporting a decrease in each corresponding income and dietary diversity measure.

Lastly, in Subsection 4.3 we further assess whether decreases in HDDS can be associated with demand‐ and/or supply‐side indicators. We accordingly augment the previous linear regression model to

(2) $$D_{ij}=\alpha +I_{ij}\delta +X_{ij}\beta +c_{j\;}+u_{ij},$$

where the dependent variable $D_{ij}$ is an indicator variable capturing whether the household reported a decrease in the DDS (HDDS, ASF, and F&V) between the two survey periods; and $D_{ij}$ is a vector of multiple demand‐ and supply‐side factors that include indicator variables for reported decreases in each income source (agricultural, nonagricultural, and remittances), whether the community was closed or had restricted entry or exit during the month prior to the survey, and whether the community received public or private aid; whether the household reported a decrease in food availability in local markets; and whether the household reported a price increase in any of the following five food groups: grains and cereals, roots and tubers, F&V, meats, and dairy products. In the case of ASF and F&V scores, we additionally consider a specification incorporating only a price increase in meats and/or dairy products and F&V, respectively.

The parameters of interest in Equation (2) are captured in the vector ϑ, which estimates the partial correlations between the demand‐ and supply‐side variables and the likelihood of reporting a decrease in each DDS. The household characteristics and location fixed effects control in this framework for potential confounding effects of household‐specific and other unobserved factors.^22^

### 3.1 Seasonality

We rely on a panel of households followed over time and compare the situation of these households at different periods within a year. In addition, our study population is rural and mostly dependent on agricultural activities. It is then relevant to discuss any potential seasonal patterns around income, food availability and prices, and dietary diversity, and assess how these could be affecting our results.

First, we analyze seasonality in income. As indicated above, a large proportion of our study sample are coffee producers, a crop harvested between October and April. To better understand these producers’ cash flow dynamics, we conducted key informant interviews with experts from coffee value chains in the area. The findings from these interviews indicate that the income from selling coffee reach farmers with a few months of delay, once the proceeds from the coffee exports become available. In particular, the informants indicated that the months in which coffee producers have the highest liquidity are generally April through June. In this sense, seasonality in cash inflow from one of the main agricultural activities for the majority of households in our sample does not seem to be a likely candidate for the lower observed dietary diversity around May–June as compared to November–December.

Second, in terms of seasonality in food availability, we note that multiple crops, including a wide set of F&V, are typically harvested at different months over the year across the country ( Ministerio de Agricultura, Ganaderia y Alimentacion, 2020). In particular, different food items within each of the broad food groups considered to construct the DDS are readily available year‐round. As a result, seasonal crop calendars should not have a major effect on the availability of foods within the groups used to derive DDS. It is important to note, though, that the region experiences a relative scarcity of foods—especially basic grains—starting on April, which becomes more critical in July through August ( Famine Early Warning Systems Network, 2016; Programa Mundial de Alimentos, 2014).^23^ This scarcity, however, is mostly relevant for the poorest households, which are relatively underrepresented in our sample, and for atypical, dry weather years.

Third, to assess seasonality in food prices, we obtained monthly average wholesale prices since January 2015 from the Ministry of Agriculture online web portal, for eight foods commonly consumed by households in the study area, namely black beans, maize, potatoes, onions, tomatoes, squash, and chicken and cow meat.^24^ Despite the large variability in prices between years, we do not observe any clear patterns in the price differences of the above foods between the months prior to the lockdown (i.e., January–February, which is the base period for self‐reported price changes) and the months in which the follow‐up survey was conducted (May–June). This suggests that such dimension does not seem to be a first order concern for our analyses.^25^

We note, however, that dietary diversity is ultimately an outcome from the interplay between income, food availability, and food prices. As a result, seasonality in dietary diversity depends on the extent of seasonal patterns around the three dimensions above and on their relative importance in determining households’ diets. To further assess whether seasonality in dietary diversity may be a concern for the validity of our findings, we calculated HDDS, WDDS, ASF, and F&V scores using secondary data from the 2018 latest available Integrated Reproductive Health and Nutrition Surveillance System surveys, fielded year‐round in Guatemala across a nationally representative sample.^26^ We find that all the DDS are, on average, higher during May–June 2018 than during November–December 2018 across all available households in Quiche and Huehuetenango.^27^ This is indicative that seasonality, if any, moves in the opposite direction to our findings below around dietary diversity patterns before and after the lockdown.

Overall, while we recognize that we cannot fully discard seasonal effects as confounding factors in our analyses, any seasonal trends in food availability and food access (income and prices), and ultimately dietary diversity, seem to operate opposite to the observed effects of the lockdown described below, providing additional support to our empirical approach.

## 4 RESULTS AND DISCUSSION

This section presents and discusses the results of the study. First, we discuss before–after comparisons on income, food security, and dietary diversity measures of interest. Second, we characterize the type of households in our sample most likely to report a decrease in their income and dietary diversity outcomes. Finally, we further examine whether the decrease in dietary diversity outcomes is correlated with demand‐ and/or supply‐driven factors.

### 4.1 Before–after comparisons

**FIGURE 2:**
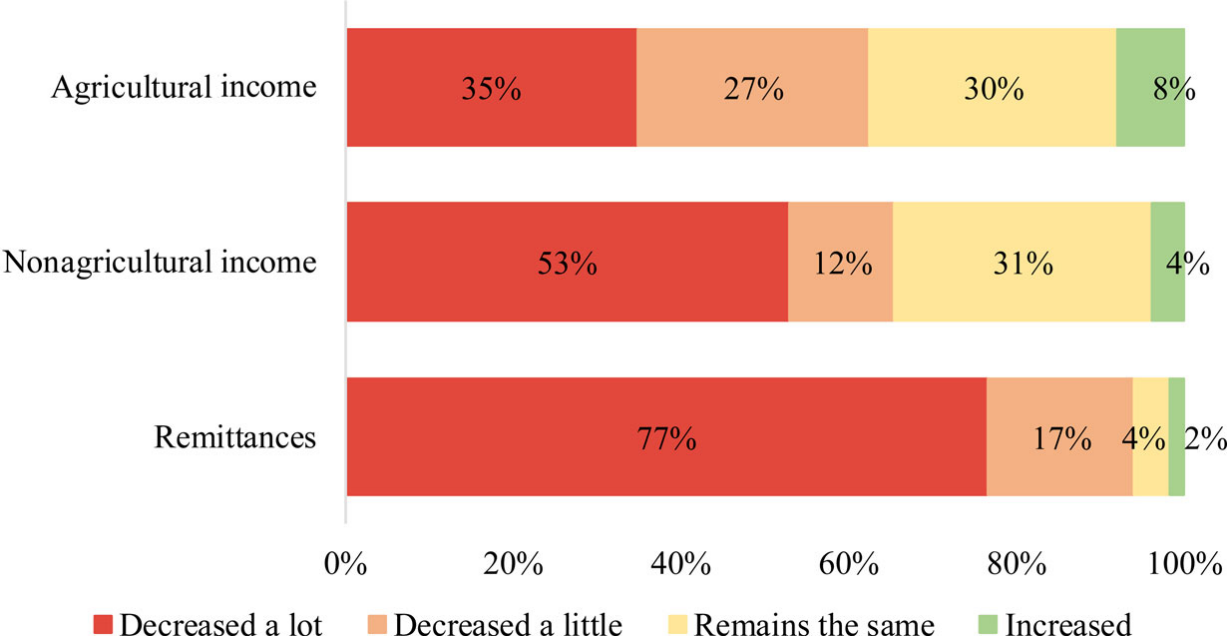
Reported changes in income sources [Color figure can be viewed at wileyonlinelibrary.com] *Note*: This figure shows the percentage of interviewed households that reported decreases or increases to their agricultural, nonagricultural, and remittances income after the lockdown. Answer categories in the questionnaire were symmetric, with households being able to declare that their income had increased either a lot or a little; however, these are lumped together in a single “Increased” category for clarity of exposition. The percentages for agricultural and nonagricultural income are based on all 1,824 households, while the percentages for remittances are only based on the subsample of 578 households that reported receiving remittances at baseline (November–December 2019) and/or the follow‐up (May–June 2020) survey

Figure 2 shows self‐reported qualitative changes in household income by source between November–December 2019 and May–June 2020, in the context of the COVID‐19 restriction measures. Almost two thirds of households report a decrease in the income earned from farm‐related activities (with 35% and 27% of the households reporting, respectively, a large and small decrease), while only 8% of the households report an increase. In the case of nonagricultural income, a similar number of households reports a decrease, though a higher proportion (53%) indicates a large decrease (with 12% reporting a small decrease). Considering that agricultural activities were exempted from the government restrictions, it is not surprising that the share of households reporting a large decrease in agricultural income is smaller compared to other income sources.^28^ The reported variations in remittances are even sharper, consistent with national reports during the first months after the outbreak. Among the households receiving remittances at baseline and/or follow‐up (578 households), an overwhelming 94% of them report a decrease in the amount received (most of them characterizing the decrease as large); and 97% also indicate a decrease in the frequency of remittances.^29^

On aggregate, roughly four out of every five households in the sample (1,428 households) report an unambiguous decrease in income, indicating a decrease in at least one of the three income sources and no increase in others.^30^ Online Appendix Figure A.1 illustrates how these households are coping with these income losses. About one third of them report that they are using their own savings to cope with the crisis, while 30% indicate that they have been selling assets (mainly livestock). More than a quarter of households, though, indicate that they are not resorting to any strategy to cope with their income losses. Interestingly, a very small percentage report borrowing from family and friends, consistent with the failure of coping mechanisms that smooth consumption across households in the face of a systemic shock (Townsend, 1994). Lastly, 23% indicate that they have been receiving aid and assistance from the government and/or private organizations; at the community level, roughly 60% of the leaders reported that the community received some form of aid or assistance (mainly public) in the month prior to the follow‐up survey, which suggests that the aid provided during the first months after the start of the pandemic has targeted multiple communities in the Western Highlands but has been focalized within each community.

Online Appendix Figure A.2 shows reported changes in food availability (panel A) and prices of different food groups (panel B) at the local market. Nine of every ten households indicate a decrease in food availability. In terms of prices, the majority of households indicate increased prices across all food groups since the start of the lockdown (69% of households report an increase in meat prices, around 80% an increase in F&V and roots and tubers, and 91% an increase in grains and cereals as well as dairy products). Both the lower food availability and uniform price increase are probably associated with the fact that 78% of the sampled communities were closed or had restricted access during the month prior to the survey (as reported by community leaders).

**FIGURE 3:**
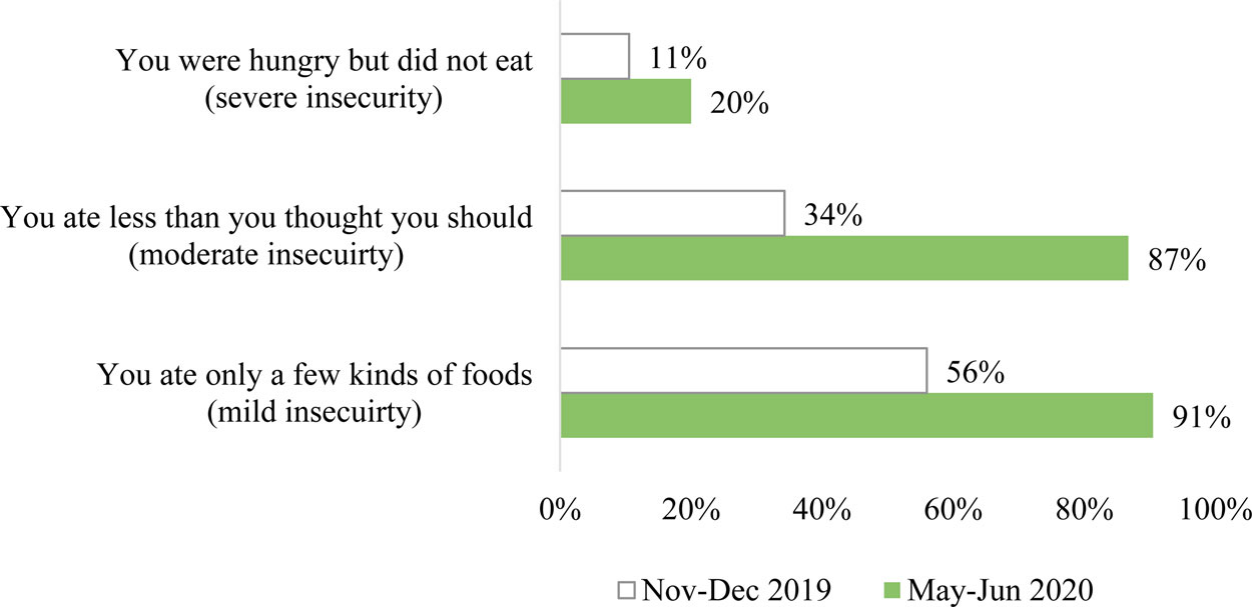
Before and after food insecurity experiences [Color figure can be viewed at wileyonlinelibrary.com] *Note*: This figure shows the percentage of 1,824 interviewed households that reported having experienced the situations described above (over the months prior to the survey), before and after the lockdown. These situations are part of the eight Food Insecurity Experience Scale items proposed by Ballard et al. (2013) and are linked to a mild, moderate, and severe level of food insecurity. The white bars correspond to the period of November–December 2019 and the green bars to the period of May–June 2020

In line with these reported changes in income, food availability, and prices, Figure 3 reveals a significant increase in the share of households reporting a food insecurity experience in the months after the lockdown, compared to the end of 2019. After the lockdown, 91% of households report having eaten only a few kinds of foods because of a lack of money or other resources (mild food insecurity), 87% report having eaten less than they thought they should (moderate food insecurity), and 20% report having not eaten despite feeling hungry (severe food insecurity), compared to 56%, 34%, and 11%, respectively, in November–December 2019. It is clear that the lockdown increased the prevalence of food insecurity among the sampled households, both in terms of inadequate diversity of foods and insufficient food quantity.

In what follows, we delve deeper into some of the nutritional impacts of the lockdown by exploring in more detail changes in various dietary diversity measures. Panel A of Table 2 reports the average HDDS, ASF, and F&V scores for the reference household member, before and after the lockdown. Several interesting patterns emerge from the table. First, we observe a statistically significant decrease in the HDDS from 6.8 (out of twelve possible food groups) to 6.4, suggesting a general decrease in the dietary diversity of households.^31^ Second, the decrease in dietary diversity seems to be mainly driven by a reduced diversity in animal proteins consumed, with an average score decrease from 1.3 (out of seven possible ASF groups) to 0.8; that is, if prior to the lockdown a household member was barely consuming a bit more than one animal protein group a day, after the outbreak they report consuming less than one group. Third, households seem to be compensating for the lower diversity in their consumption of animal proteins with a higher diversity in their consumption of F&V, with the average F&V score increasing from 2.1 (out of five possible F&V groups) to 2.5. While we did not collect information regarding specific availability of food items in local markets, as noted earlier multiple F&V are typically available year‐round and it is likely that households resorted to expanding their consumption of different locally available F&V.^32^

**TABLE 2:** Before and after average comparisons of dietary diversity indicators

Indicator	November–December 2019	May–Jun 2020	Difference *p*‐value
Panel A: Household level (1,824 observations)			
Dietary Diversity Score (HDDS)	6.848	6.440	0.000
	(1.862)	(1.582)	
Animal source food (ASF) consumption score	1.290	0.821	0.000
	(1.007)	(0.781)	
Fruits and vegetables (F&V) consumption score	2.145	2.513	0.000
	(1.461)	(1.345)	
Panel B: Women 15–49 years old (1,603 observations)			
Dietary Diversity Score (WDDS)	4.555	4.487	0.149
	(1.427)	(1.260)	
ASF consumption score	1.279	0.810	0.000
	(1.011)	(0.774)	
F&V consumption score	2.134	2.509	0.000
	(1.465)	(1.354)	
Panel C: Children 6–23 months old (318 observations)			
Dietary Diversity Score (DDS)	3.286	3.899	0.000
	(1.659)	(1.211)	
ASF consumption score	0.909	0.786	0.078
	(0.944)	(0.805)	
F&V consumption score	1.503	2.189	0.000
	(1.509)	(1.430)	

*Note*: This table reports the average and standard deviation (in parentheses) of the dietary diversity indicators, before and after the lockdown. “Difference *p*‐value” results from the mean‐comparison *t*‐test between the two periods (assuming unequal variances) where a value larger than 0.05 indicates that the average difference in each variable across the two periods is not statistically different at a 95% confidence level. The HDDS, ASF, and F&V scores measure the number of food groups that a member of the household consumed over the previous 24 h, while the WDDS and children DDS measure the number of food groups that a selected woman 15–49 years old or a child 6–23 months old in the household consumed over the previous 24 h. (See the main text for the groups comprised in each of the scores.)

**FIGURE 4:**
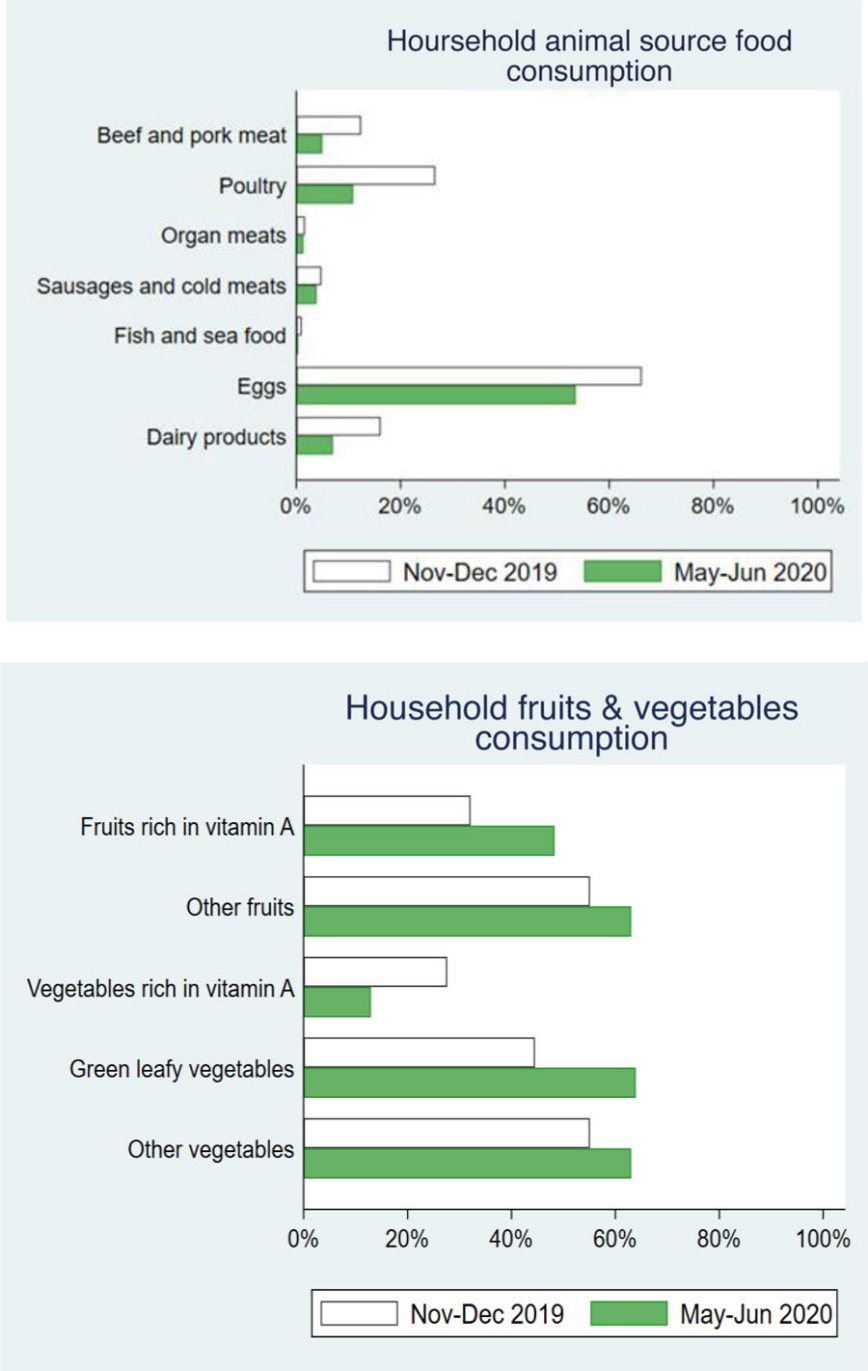
Before and after household consumption of ASF and fruits & vegetables [Color figure can be viewed at wileyonlinelibrary.com] *Note*: This figure shows the percentage of households reporting having consumed each of the food groups over the previous 24 h, before and after the lockdown. The total number of surveyed households is 1,824. The white bars correspond to the period of November–December 2019 and the green bars to the period of May–June 2020

Figure 4 provides additional insights about the observed changes in the consumption of ASF and F&V groups. The figure shows the percentage of households that report consuming each ASF group and each F&V group before and after the lockdown. We find an overall decrease across all ASF groups, including eggs and poultry—which are the usual animal proteins consumed in the area—followed by dairy products and beef and pork meat. In the case of F&V, we observe an increase across all categories except for a decrease in consumption of vegetables rich in vitamin A. Hence, if households in the region were already not including much ASF in their diets, the lockdown seems to have further aggravated this pattern. However, the shift towards a higher diversity in the consumption of F&V implies a higher intake of nutrient‐rich foods. All in all, although the nutrient bioavailability from F&V is lower than from ASF (Allen & Gillespie, 2001; Murphy & Allen, 2003), it is hard to determine the net change in nutrients intake from our data alone, without considering quantities consumed.^33^

Turning to the dietary diversity indicators for women and children, panels B and C of Table 2 present the corresponding dietary diversity average scores for a selected woman in the household of 15–49 years old and a child 6–23 months old, before and after the lockdown. The number of households with women and children are, respectively, 1,603 and 318.^34^

In the case of women, we find no substantial changes in the DDS (WDDS) since the lockdown. The average score remains around 4.5 (out of nine possible food groups). When disaggregating this score, the diversity of ASF consumption among women seems to have decreased and the diversity of F&V consumption increased (see Online Appendix Figure A.4).^35^ In contrast to the HDDS and WDDS, the children DDS shows an average score increase from 3.3 (out of seven possible food groups) to 3.9. In this case, while diversity in overall ASF consumption marginally decreased (with an increase in the share of children consuming eggs, sausages, and cold meats), diversity in F&V consumption increased significantly.^36^

In sum, the COVID‐19 lockdown seems to have affected the incomes, food security, and dietary patterns of rural households in the Western Highlands, with a decrease in ASF diversity and an increase in F&V diversity, and an overall net decrease in diversity across all food groups. Dietary diversity among women, however, appear to have remained unchanged, while dietary diversity among children increased. This apparent prioritization of young children suggests potential intrahousehold reallocation of foods in response to external shocks, documented in previous studies among vulnerable, rural populations (see, e.g., Block et al., 2004; Skoufias & Vinha, 2012).^37^ This is an important aspect that should be explored more carefully in future research, especially in a context of limited risk coping mechanisms. In terms of potential effects on dietary quality, it is unclear whether the substitution in diversity away from ASF and into F&V implies overall changes in protein and micronutrient intake, considering the lower nutrient bioavailability in F&V and the importance of certain animal proteins in child growth (Arimond & Ruel, 2004; Food and Nutrition Technical Assistance III Project, 2015; Krebs et al., 2011).^38^ Since we do not count with more detailed food consumption data to assess nutrient intake, such an analysis is beyond the scope of this article.

### 4.2 Profile of households reporting a decrease in income and dietary diversity

**FIGURE 5:**
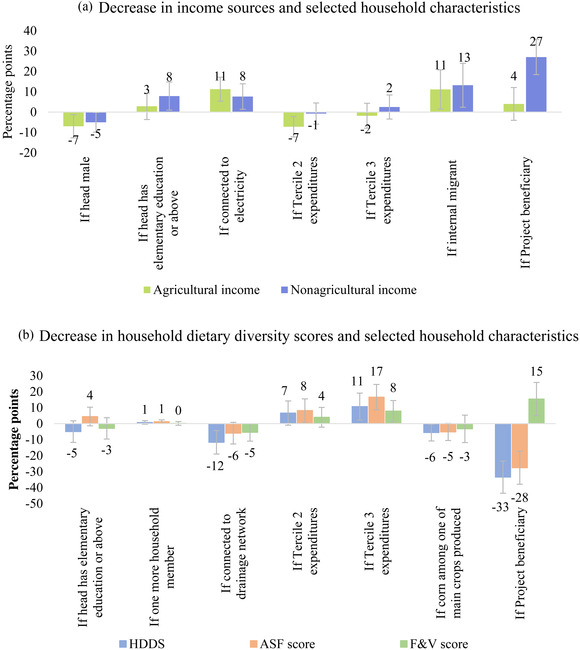
Partial regression coefficients between a decrease in income and dietary diversity and household characteristics. (a) Decrease in income sources and selected household characteristics. [Color figure can be viewed at wileyonlinelibrary.com] (b) Decrease in HDDS and selected household characteristics *Note*: This figure shows the partial correlations (in percentage points) of regressing if the income source or DDS decreased after the lockdown on a set of household characteristics at baseline, for selected variables. The vertical lines correspond to the 95% confidence intervals. The full estimation results are reported in Online Appendix Tables A.2 and A.3. The correlations in both panels are based on 1,824 surveyed households. The HDDS, ASF, and F&V scores measure the number of food groups that a member of the household consumed over the previous 24 h. (See the main text for the groups comprised in each of the scores.)

We now turn to explore the main characteristics of the households in our sample that report decreases in incomes and dietary diversity following the regression framework depicted in Equation (1). For clarity of exposition, Figure 5 shows selected (mostly significant) coefficients for relevant household variables from the full regressions modeling a decrease in income sources (Figure 5a) and HDDS (Figure 5b).^39^ From Figure 5a, we observe that female‐headed households in our sample with a migrant worker at home as well as households with access to electricity (more prevalent in San Marcos) are more prone to report a decrease in both agricultural and nonagricultural income after the lockdown. Similarly, households with more educated heads and beneficiaries of the Value Chains Project are more likely to show a decrease in nonagricultural income. Regarding differences by income level (proxied by per capita expenditures at baseline), it appears that households in the middle tercile were less affected than households in the lower and upper terciles.^40^

Figure 5b shows that the decrease in most household dietary diversity indicators is positively correlated with per capita expenditures at baseline suggesting that richer households have on average decreased their dietary diversity more than poorer households (although richer households still consume more diverse foods than poorer ones). Larger households are also marginally associated with a decrease in the ASF score. Households for whom corn is one of their main crops, which are generally poorer, are similarly less likely to exhibit a decrease in total dietary diversity and ASF scores, compared to other producers. Lastly, it is interesting that beneficiaries of the Value Chains Project are more likely to show a decrease in F&V scores but less likely to show a decrease in ASF scores (and aggregate HDDS), considering that promoting the consumption of ASF is one of the main nutritional interventions of the Project, as noted earlier.^41^

To further explore the variations in dietary diversity patterns by income level, Online Appendix Figure A.5 reports local polynomial curves of HDDS, ASF, and F&V scores in 2019 (gray line) and 2020 (green line) against the natural logarithm of per capita expenditures measured at baseline. The shaded areas represent the 95% confidence bands. Note that the curves are generally upward sloping in both periods, especially for ASF and HDDS, confirming that richer households maintained more diverse diets before and after the lockdown. Yet, when comparing across periods, we effectively observe a decrease in HDDS among richer households while poorer households show a marginal (and noisier) increase. Similarly, the decrease in the ASF score is clearer and sharper among richer households, with no observable changes among poorer households. This may be explained by the fact that households with relatively higher incomes were previously consuming more poultry, dairy products, and beef and pork meat (besides eggs) and have stopped the consumption of some of these ASF groups after the lockdown. Increases in the F&V score, in turn, are more widespread across income levels.^42^

Overall, households in our sample with relatively higher income at baseline (who were also consuming more diverse foods) seem to have been more likely to experience a decrease in their dietary diversity after the lockdown. These households, which are also more prone to report a decrease in income (especially on the upper end of the income level distribution and in San Marcos), could have been affected to a larger extent by the lockdown and other measures related to COVID‐19 (including the temporal closure of some communities) than relatively poorer households, who tend to depend more on subsistence farming and other small scale, locally oriented activities, and probably experienced less disruptions. In a similar vein, we would expect very poor farmers, which are underrepresented in our study sample, to have been relatively less affected, similar to households in the lower end of the income distribution in our sample. Yet, these populations may still be worse off in absolute terms and exhibit other vulnerabilities along several dimensions. Acute malnutrition, for example, which is connected to a decrease in food consumption and/or illnesses derived from lack of potable water and poor sanitation, more than doubled in Guatemala over the months after the start of the pandemic compared to same period in 2019.^43^

### 4.3 Association between a decrease in dietary diversity and demand and supply factors

Finally, we assess whether the observed decrease in HDDS can be associated with demand‐ and/or supply‐side indicators. Table 3 presents the partial regression coefficients between the decrease in HDDS, ASF, and F&V scores and a set of factors described in Equation (2).

**TABLE 3:** Partial regression coefficients between a decrease in household dietary diversity and demand and supply factors

	(1)	(2)	(3)	(4)	(5)
Coefficient	If HDDS decreased	If ASF score decreased	If ASF score decreased	If F&V score decreased	If F&V score decreased
If decrease in agricultural income	−0.011	−0.017	−0.016	−0.011	−0.013
	(0.031)	(0.034)	(0.034)	(0.026)	(0.026)
If decrease in nonagricultural income	0.053*	0.025	0.025	0.043	0.044
	(0.031)	(0.023)	(0.024)	(0.034)	(0.034)
If decrease in remittances	0.057**	0.010	0.010	0.035	0.031
	(0.027)	(0.024)	(0.025)	(0.026)	(0.026)
If community restricted access	0.354**	0.349	0.344	0.152	0.146
	(0.142)	(0.392)	(0.396)	(0.225)	(0.221)
If community received aid	0.047	−0.139	−0.139	−0.085	−0.090
	(0.056)	(0.218)	(0.220)	(0.101)	(0.101)
If decrease in food availability	0.070*	0.010	0.011	0.013	0.008
	(0.038)	(0.050)	(0.050)	(0.038)	(0.037)
If increase in food prices	−0.004	0.004		0.021**	
	(0.009)	(0.010)		(0.010)	
If price increase in meats and/or dairy products			−0.002		
			(0.018)		
If price increase in fruits and vegetables					0.110***
					(0.034)
Observations	1,824	1,824	1,824	1,824	1,824

*Note*: This table reports the regression results of estimating by ordinary least squares if the dietary diversity score decreased after the lockdown on a set of demand‐ and supply‐side indicators, while controlling for household characteristics and location fixed effects. Robust standard errors reported in parentheses clustered by community.

^***^ *p* < 0.01, ^**^ *p* < 0.05, ^*^ *p* < 0.1. The dependent variable is equal to one if the household shows a decrease in the corresponding dietary diversity indicator, and zero otherwise. The HDDS, ASF, and F&V scores measure the number of food groups that a member of the household consumed over the previous 24 h. (See the main text for the groups comprised in each of the scores as well as for the description of the demand‐ and supply‐side indicators.)

We find a positive correlation between restricted community access and a lower dietary diversity. In particular, households located in communities that constrained their entry or exit, which likely affected the functioning of local economies, commercialization, and sales, show a 35.4 percentage points higher probability of reporting a decrease in HDDS (for ASF and F&V scores the correlation is highly positive but not statistically significant). Similarly, households that experienced a decrease in income from remittances exhibit a 5.7 percentage points higher probability of reporting a lower HDDS, while households indicating lower food availability in local markets show a 7 percentage points higher probability of reporting a lower HDDS. A decrease in nonagricultural income is also positively associated with a lower dietary diversity, while price variations do not seem correlated with changes in dietary diversity (except for F&V). We also do not find evidence that households located in communities that received some form of aid are less likely to report a decrease in dietary diversity. Overall, these findings, and particularly those for the HDDS, provide additional support to attribute the observed changes to the COVID‐19 lockdown, through both demand and supply channels.^44^

## 5 CONCLUDING REMARKS

We assess the short‐term effects of COVID‐19 on food security and nutrition in the Western Highlands of Guatemala. This region is possibly the most vulnerable area in the country, with poverty and chronic malnutrition rates of up to 81% and 63%, respectively. We focus on variations in income sources and food consumption patterns and rely on two waves of a panel dataset of 1,824 small agricultural households, collected shortly before and after the lockdown and other restrictions came into effect.

The large majority of the households interviewed self‐report a decrease in their agricultural and nonagricultural income and in their income from remittances. Households report either accessing their savings, selling assets (particularly livestock), or relying on assistance from the government or other organizations as their main coping mechanisms to this negative income shock. In addition, most households report reduced food availability in local markets and increased prices across a range of food groups, which combined with lower earnings appear to have contributed to a higher prevalence of food insecure experiences after the lockdown. In terms of dietary diversity, we find a reduction in average HDDS, in contrast to women and young children DDS that seem to have remained unchanged or increased, respectively, after the lockdown. When decomposing these effects, we observe a reduction in the number of ASF groups consumed, compensated by an increase in the number of F&V groups consumed that is more salient among children. No significant changes are found in other food groups such as cereals and grains or legumes and nuts, which are major components of households’ diets in the area. Lastly, households with relatively higher income levels at baseline appear to have reduced their dietary diversity to a larger extent than lower income ones, as well as households located in communities that imposed more stringent access restrictions due to the pandemic.

Our study highlights some important dimensions to be considered when designing policy responses to the pandemic. We report decreases in households’ food security and in overall dietary diversity following reductions in income, price increases, and lower food availability at local markets. Except for children under 2 years old (and, to a lower extent, women in reproductive age), other household members appear to have reduced their dietary diversity. Since dietary diversity has been associated to nutrient adequacy in both children and adults (Bianchi et al., 2016; Foote et al., 2004; Ruel et al., 2013; Steyn et al., 2014), our results could be of concern in a nutritionally compromised context such as Guatemala's Western Highlands. If there are micronutrient deficiencies, these can impair immune function (Calder, 2013), further raising the vulnerability of these populations to the virus.

In this sense, direct measures to supplement household incomes and ensure an adequate supply of different types of food in local markets, particularly in communities that have imposed more severe access restrictions, are important to minimize the medium‐ and long‐term effects of the COVID‐related crisis. Starting in April 2020, the Government of Guatemala put in place several measures intended to contain the negative effects of the crisis on livelihoods and food security, including programs to support micro, small, and medium enterprises, subsidies for public services, and price controls of foods included in the basic food basket.^45^ Closer to our study population, the government launched the “Programa de Apoyo Alimentario,” a nationwide program to distribute food rations to vulnerable urban and rural families prioritizing the procurement of basic grains from smallholder farmers. Similarly, the “Bono Familia” was launched providing a temporal supplementary monthly income of 130 U.S. dollars to vulnerable families with a monthly electricity consumption below 200 kWh (based on their electricity bill); such program, however, has received criticisms due to mostly excluding families without access to electricity or relying on digital platforms for enrollment in a context of large digital access gaps (Paraíso Desigual et al., 2020).^46^ Despite the advances of these programs, our data show, for example, that while six out of every ten communities received some form of public or private aid (as reported by the leaders), only two out of every ten households reported receiving aid when asked about it in the survey, suggesting the need to further intensify current efforts to reach a larger share of rural households that have been affected by COVID‐19. Lastly, we point out the importance of devising an appropriate mechanism to allow for the internal migration of temporary laborers for the upcoming harvest season of multiple export crops, both to ensure the availability of labor for harvest and because such work also entails an essential source of income for many rural households in Guatemala.

Finally, while we rely on a rich panel dataset of households with information collected prior and after the lockdown, we certainly cannot fully discard other unobserved factors affecting the results as the analysis is based on before–after comparisons. As noted above, the reported changes in income sources after the lockdown could be subject to a negative perception bias, although this source of bias would become more relevant when trying to distinguish between small and large (qualitative) decreases in income. Similarly, while we cannot rule out seasonal variations in the availability and accessibility of certain foods, secondary evidence on variations in dietary diversity in the study region over the months of interest indicate that a potential seasonality bias, if any, would go in the opposite direction to our findings. Future work includes additional follow‐up surveys towards the end of 2020 and 2021 to assess longer term variations on food security and nutritional patterns in the region, including anthropometric measures.

## Supporting information

Figure A.1. Reported coping mechanisms for income losses
Figure A.2. Reported changes in local food availability and prices
Figure A.3. Before and after distribution of household dietary diversity indicators
Figure A.4. Before and after consumption of ASF and fruits & vegetables for women 15‐49 years old and children 6‐23 months old
Figure A.5. Before and after local polynomial plots of household dietary diversity indicators by income level
Table A.1. Orthogonality test between households included and not included in the analysis
Table A.2. Regressions if income decreased on household baseline characteristics
Table A.3. Regressions if dietary diversity decreased on household baseline characteristics

Supplementary Material

## Footnotes

1 As of mid‐September 2020, the index was 87.04 and there were still several restrictions in place as Guatemala had not yet entered the COVID‐19 recovery phase (planned in four stages).

2
https://www.banguat.gob.gt/ (accessed on September 2020). Remittances started to recover though since May 2020. It is worth noting that Guatemala is the second country in Latin America (after Mexico) in terms of family‐related remittances, amounting to 10,508.3 million US dollars in 2019, or 13.8% of the GDP.

3 The program is Feed the Future Guatemala Value Chains Project funded by the U.S. Agency for International Development (USAID) and implemented by Agropecuaria Popoyan and Federacion de Cooperativas Agricolas de Productores de Cafe de Guatemala (FEDECOCAGUA). The objective of the program is to increase agricultural incomes and resilience for small farmers and their families in the Western Highlands and improve their nutritional status by integrating ASF‐ and Social Behavior Change and Communication–related interventions into a value chain focused portfolio of activities.

4 The Western Highlands region has the highest poverty and malnutrition rates in Guatemala and is characterized by smallholder farming, low agricultural productivity, and reduced market access.

5 A cluster randomized design was pursued to obtain a representative sample of beneficiary and control households. A subset of clusters (communities, in the case of Popoyan, or farmer associations, in the case of FEDECOCAGUA) were selected from updated lists of project beneficiaries. Clusters reporting at least one household with a child under 5 years old were automatically included, and the remainder were randomly selected from the full list of communities or farmer associations, stratified by department. Once clusters had been selected, any beneficiary households with at least one child under 5 years old or women in reproductive age (including pregnant women) were directly included into the final household sample, together with a random sample from the remaining listed households in those communities, up to a maximum of 30 households per community. In order to select control households, neighboring communities with similar agroecological, socioeconomic, and market access characteristics as the treatment communities were selected and all households cultivating coffee, cardamom, or horticultural among one of their main crops, as identified in consultation with local leaders, were interviewed.

6 It is worth noting that an additional 337 households were also interviewed at baseline (in addition to the 2,142 households indicated above) but did not provide a cell phone number. These are generally poorer households, and most of them are located in Quiche, which is precisely the department with the highest poverty and chronic malnutrition rates.

7 The community leaders were also briefly interviewed to obtain some information at the community level such as whether the community was closed or had restricted entry or exit during the month prior to the survey as well as whether the community received some form of aid or assistance from the government and/or private organization.

8 Of the 318 households that were interviewed at baseline but are not part of the final sample, 57 declined to participate in the follow‐up survey, 24 did not provide information for all key variables, and 237 could not be reached by phone (mainly due to permanent low network connectivity in the area). To assess for potential sample selection, Online Appendix Table A.1 shows orthogonality tests for differences in average characteristics at baseline between the final study sample and households not included in the analysis. Overall, we find balance across most characteristics. Among the very few differences observed at the 5% level between the two groups, households in the final study sample seem to have a slightly older household head who also speaks Spanish, higher daily per capita expenditures, a smaller share of them report corn as one of their main crops grown, and a slightly smaller share did not report receiving income from agricultural activities in 2019.

9 The sample‐average household head education, dwelling materials (finished walls, ceiling, and floors), connection to electricity, and asset ownership (TV or radio) are slightly better off than the corresponding averages for a rural household in Huehuetenango, Quiche, and San Marcos derived from the 2018 Population and Housing Census (https://www.censopoblacion.gt/, accessed on September 2020).

10 The base of comparison for changes in food availability and prices is the period before the lockdown (and not the end of 2019) due to the specific nature of these questions, which could be more prone to recall errors. Certainly, self‐reported changes may still be subject to other potential biases, especially for income measures, which we discuss in the next section.

11 The remaining 6% of the total income reported in 2019 correspond to donations and transfers received by the household. The three departments covered in the study are also among the departments that receive more family remittances in the country.

12 We also included an option in the survey for “Does not have” considering that several households might not receive all income sources, particularly nonagricultural income and remittances. As such, households with no income at baseline and follow‐up are coded as no income change; similarly, those with no income at baseline but reporting an income change in the follow‐up are coded according to their reported change. In the analyses below, we combine the two “increased” categories into a single one for clarity of exposition, given that only a very small percentage of households reported increases in their incomes.

13 See Leroy et al. (2015) for a detailed review and assessment of different food access indicators.

14 It is important to note that, since the dietary diversity questions were asked for a reference member in the household, the resulting HDDS score is not the traditional HDDS, which inquires about food groups consumed by anyone in the household. Since the aim of the score is to approximate the diversity of foods consumed by an average (reference) member in the household and uses the same food groups, we still refer to it hereafter as HDDS for simplicity.

15 During the baseline survey, a reference household member, one child under 2 years old and one woman (usually the mother or caregiver of the child) were selected in each household for whom to collect the dietary diversity module. During the follow‐up survey, the modules were again aimed at these same individuals.

16 The twelve food groups in the HDDS are (1) cereals and grains; (2) roots and tubers; (3) vegetables; (4) fruits; (5) meats, poultry, and offal; (6) eggs; (7) fish and seafood; (8) legumes and nuts; (9) milk and milk products; (10) oil and fats; (11) sugar; and (12) miscellaneous foods. The nine food groups in the WDDS are (1) cereals, grains, roots, and tubers; (2) dark green vegetables; (3) other fruits and vegetables rich in vitamin A; (4) other fruits and vegetables; (5) organ meats; (6) meat, poultry, and fish; (7) eggs; (8) legumes, nuts, and seeds; and (9) milk and milk products. The seven food groups in the children DDS are (1) cereals, grains, roots, and tubers; (2) legumes and nuts; (3) dairy products (milk, yogurt, cheese); (4) flesh foods (meat, fish, poultry, and liver/organ meats); (5) eggs; (6) vitamin A rich fruits and vegetables; and (7) other fruits and vegetables.

17 The ASF food groups include (1) beef and pork meat; (2) poultry; (3) offal; (4) sausages and cold meats; (5) sea food; (6) eggs; and (7) dairy products. The F&V food groups include (1) fruits rich in vitamin A; (2) other fruits; (3) vegetables rich in vitamin A; (4) green leafy vegetables; and (5) other vegetables.

18 The results are robust to implementing a discrete choice model.

19 The working sample for the agricultural and nonagricultural income assessments is the full sample, as a large fraction of households report these income sources at baseline and/or report income changes in the follow‐up survey; hence, the (small) share of households that do not report an income in both periods are also assigned a zero value. In the case of remittances, we restrict the analysis to those households reporting remittances at baseline and/or follow up (578 households) since the vast majority in this case do no report receiving remittances during both periods.

20 Godlonton et al. (2018) document this bias among smallholder farmers in Central America.

21 Since the impact evaluation of the Value Chains Project does not have a focus on risk management around shocks, we did not measure some potentially relevant variables at baseline, so these are omitted from the analyses. For instance, households’ access to different coping mechanisms or formal risk management instruments such as insurance may be important determinants of their ability to cope with a large systemic shock such as COVID‐19. We note, however, that the penetration of formal agricultural risk management instruments in the area is negligible, and many informal risk coping mechanisms tend to fail in the presence of a systemic shock.

22 For example, self‐consumption versus bartering and purchasing food products in local markets may vary across households and locations with different characteristics, which may, in turn, affect reported changes in food availability or prices.

23 This period also shows highest prevalence of acute malnutrition in the calendar year ( Secretaría de Seguridad Alimentaria y Nutricional, 2013).

24
https://precios.maga.gob.gt/ (accessed on September 2020).

25 Details are available upon request.

26 This survey is collected by the Institute of Nutrition of Central America and Panama in coordination with the Centers for Disease Control and Prevention.

27 These are not the same households, but different households interviewed in those two time periods belonging to the same department. Despite its limitations, these data are the best available to determine the extent of seasonality in dietary diversity in the region. The results are available upon request.

28 Although we did not inquire about specific reasons for the reported decreases in income during the follow‐up survey, some of the decrease in agricultural income could be related to disruptions in the payment schedule downstream the value chain during the first months after the outbreak. In the case of coffee, for instance, there were disruptions in green coffee shipments, which could have impacted payments to processors and, ultimately, producers.

29 When disaggregating by department, we observe similar patterns of reported decreases in income across households in all three departments (Huehuetenango, Quiche, and San Marcos). Households in San Marcos, however, also report very large decreases in agricultural and non‐agricultural income (81% and 73%, respectively). This could be explained by the fact that San Marcos is relatively more developed than the other two departments and the productive and economic activities are more connected to markets, such that the lockdown and other restriction measures could have had a larger effect on the supply and distribution channels of multiple activities in this department. Households in San Marcos also exhibit a larger average per capita daily expenditure than those in Huehuetenango and Quiche. We take into account potential variations across locations in the analysis of the subsequent sections. It is also worth indicating that if we replicate Figure 2 considering only households reporting incomes at baseline, we observe a similar pattern of decreasing incomes (35% report a large and small decrease in agricultural income, 74% in non‐agricultural income, and 77% in remittances).

30 The number of households reporting an ambiguous change in income is 92, or 5% of the total sample.

31 Online Appendix Figure A.3 shows the corresponding histograms with the distribution of the dietary diversity scores at baseline (white bars) and follow‐up (green bars) where we observe a clear left shift in the distribution of the HDDS. There is a decrease in the share of households reporting consuming eight through eleven food groups and an increase in the share consuming five through seven food groups.

32 We observe a larger increase in F&V diversity among households located in communities that restricted their access, which also points that F&V supply shortages have not necessarily been an issue in the region. Below we also find a positive correlation between households reporting a decrease in F&V prices in their local markets and an increase in the F&V diversity score.

33 Although not reported, the share of households consuming cereals and grains (corn) and legumes and nuts (black beans) was either the same or slightly increased after the lockdown: all households (100%) report consuming cereals and grains in both periods, while 80% report consuming legumes in 2020 versus 71% in 2019.

34 It is worth noting that the HDDS, ASF, and F&V variations for the reference household member in these subsamples of households are similar to the full‐sample results described earlier.

35 Similar to the patterns observed for the representative household member, there is a uniform decrease in the percentage of women consuming each of the ASF groups and a uniform increase in all F&V categories, except for vegetables rich in vitamin A.

36 As shown in Online Appendix Figure A.4, the share of children consuming all F&V categories but vegetables rich in vitamin A increased, particularly green leafy vegetables (e.g., chard, spinach, broccoli).

37 As a robustness check, we calculated dietary diversity scores for children and women but using the same groups of the HDDS (instead of the different set of groups in the children DDS and WDDS). We observe an increase in dietary diversity among children and a decrease among women, further supporting a potential change in intrahousehold food allocation.

38 The share of households reporting consumption of starchy staples among women in the household is 100% in both periods, while the share reporting women having consumed legumes increased from 70% in 2019 to 80%. Among children, consumption of starchy food and legumes increased, respectively, to 99% (from 88%) and to 81% (from 52%).

39 The full regression results are reported in Online Appendix Tables A.2 (income outcomes) and A.3 (dietary diversity outcomes).

40 Online AppendixTable A.2 also reports the results for remittances income among households that reported remittances at baseline and/or follow‐up, although we do not find statistically significant correlations other than beneficiaries of the Value Chains Project and coffee producers exhibiting a lower likelihood of reporting a decrease in remittances. This is probably explained by the fact that changes in remittances are more associated with characteristics of the migrant and the situation at the destination location than with characteristics of the household left behind.

41 Online Appendix Table A.3 includes the regression results for WDDS and children DDS. The estimated correlations for WDDS are very similar to the HDDS correlations, while in the case of children a lower educational achievement by the household head is highly correlated with a decrease in the dietary diversity score among children.

42 Although not reported, the before and after local polynomial curves for WDDS show a similar pattern as the HDDS, with a decrease in 2020 among richer households and a noisier increase among poorer households. In the case of the curves for children DDS, we observe a general increase in 2020 across all income levels, though the differences are not significant at the lower and higher ends of the income distribution. Details are available upon request.

43
http://www.siinsan.gob.gt/siinsan/desnutricion‐aguda/ (accessed on September 2020).

44 We estimated an alternative model using the difference in the dietary scores between 2020 and 2019 as the dependent variable (instead of a dichotomic variable) and we find qualitatively similar correlation patterns. Households in closed communities show, for instance, a 1.8 lower HDDS than households in communities that remained open. We also considered an alternative specification including an indicator variable that identifies households with an unambiguous decrease in income (instead of three separate indicator variables by income source), but we do not find a statistically significant correlation between this indicator and a decrease in dietary diversity.

45
https://www.congreso.gob.gt/detalle_pdf/decretos/13517 (accessed on September 2020).

46 For instance, 15% of the households in our sample do not have a dwelling connected to electricity. While the program allows for a grievance mechanism for those households without an electricity bill, education and awareness are still important obstacles to receive program benefits among this relatively disadvantaged group of households.
