# Supplementary material for: Short‐term impacts of COVID‐19 on food security and nutrition in rural Guatemala: Phone‐based farm household survey evidence
Source: Agric Econ. 2021 May 2;52(3):477–94. doi: 10.1111/agec.12629 (PMC8206951; doi:10.1111/agec.12629)
Supplement: Supplementary file 1 — Figure A.1. Reported coping mechanisms for income losses Figure A.2. Reported changes in local food availability and prices Figure A.3. Before and after distribution of household dietary diversity indicators Figure A.4. Before and after consumption of ASF and fruits & vegetables for women 15‐49 years old and children 6‐23 months old Figure A.5. Before and after local polynomial plots of household dietary diversity indicators by income level Table A.1. Orthogonality test between households included and not included in the analysis Table A.2. Regressions if income decreased on household baseline characteristics Table A.3. Regressions if dietary diversity decreased on household baseline characteristics [file AGEC-52-477-s002.docx]

**Assessing the short-term impacts of COVID-19 on food security**

**and nutrition in rural areas: Evidence from Guatemala**

**Online Appendix**

**Francisco Ceballos^1^ | Manuel A. Hernandez^1^ | Cynthia Paz^1^**

^1^Markets, Trade and Institutions Division, International Food Policy Research Institute (IFPRI), Washington, DC, USA

**Correspondence**

Manuel A. Hernandez, Markets, Trade and Institutions Division, International Food Policy Research Institute (IFPRI), Washington, DC 20005, USA.

Email: [m.a.hernandez@cgiar.org](mailto:m.a.hernandez@cgiar.org)

**Figure A.1. Reported coping mechanisms for income losses**

Note: This figure shows the percentage of interviewed households that reported using different coping mechanisms as a result of a decrease to their agricultural, non-agricultural, or remittances income after the lockdown. The values are based on the sample of 1,280 households that unambiguously reported a decrease in at least one income source and answered the questions on coping mechanisms.

**Figure A.2. Reported changes in local food availability and prices**

Note: This figure shows the percentage of interviewed households that reported changes in food availability (Panel A) and changes in food prices (Panel B) at their local market after the lockdown. The values are based on the sample of 1,824 households.

**Figure A.3. Before and after distribution of household dietary diversity indicators**

Note: This figure shows the distribution of the HDDS, ASF, and V&F scores before and after the lockdown across 1,824 surveyed households. The white histogram bars correspond to the period of November-December 2019 and the green bars to the period of May-June 2020. The vertical dotted red line indicates the average score in 2019 and the solid red line the average score in 2020. These dietary diversity scores measure the number of food groups that a member of the household consumed over the previous 24 hours. See the main text for the groups comprised in each of the scores.

**Figure A.4. Before and after consumption of animal source foods and fruits & vegetables for women 15-49 years old and children 6-23 months old**

Panel A: Animal Source Food (ASF) consumption

Panel B: Fruits & Vegetables (F&V) consumption

Note: This figure shows the percentage of households with a selected woman 15-49 years old or selected child 6-23 months old, reporting having consumed each of the food groups over the previous 24 hours, before and after the lockdown. The percentages are based on 1,603 surveyed households for the case of women and on 318 surveyed households for the case of children. The white bars correspond to the period of November-December 2019 and the green bars to the period of May-June 2020.

**Figure A.5.** **Before and after local polynomial plots of household dietary diversity indicators by income level**

Note: This figure shows local polynomial plots between the HDDS, ASF, and V&F scores and the natural logarithm of per capita daily expenditures, before and after the lockdown across 1,824 surveyed households. The local polynomial plots are derived using an Epanechnikov kernel, standard rule-of-thumb bandwidth, and assume a polynomial of degree two. The shaded areas represent the 95% confidence bands. These dietary diversity scores measure the number of food groups that a member of the household consumed over the previous 24 hours. See the main text for the groups comprised in each of the scores.

**Table A.1. Orthogonality test between households included and not included in the analysis**

| **Characteristics at baseline (Nov-Dec 2019)** | **Households** | **Households not** | **p-value** |
| --- | --- | --- | --- |
|  | **included in** | **included in** |  |
|  | **analysis** | **analysis** |  |
| If household head is male | 0.833 | 0.814 | 0.423 |
|  | (0.012) | (0.024) |  |
| Household head age | 48.049 | 44.965 | 0.010 |
|  | (0.697) | (1.247) |  |
| If household head has no education | 0.351 | 0.393 | 0.274 |
|  | (0.020) | (0.044) |  |
| If household head did not complete elementary education | 0.318 | 0.277 | 0.202 |
|  | (0.019) | (0.034) |  |
| If household head completed elementary education or above | 0.331 | 0.330 | 0.974 |
|  | (0.018) | (0.034) |  |
| If household head main language spoken is Spanish | 0.320 | 0.204 | 0.016 |
|  | (0.054) | (0.061) |  |
| Household size | 5.764 | 5.818 | 0.801 |
|  | (0.118) | (0.217) |  |
| If household is beneficiary of social school program | 0.273 | 0.289 | 0.585 |
|  | (0.018) | (0.031) |  |
| If dwelling has finished walls | 0.479 | 0.403 | 0.216 |
|  | (0.032) | (0.072) |  |
| If dwelling has finished ceiling | 0.143 | 0.129 | 0.587 |
|  | (0.016) | (0.030) |  |
| If dwelling has finished floor | 0.561 | 0.462 | 0.115 |
|  | (0.034) | (0.074) |  |
| If dwelling is connected to electricity | 0.850 | 0.726 | 0.226 |
|  | (0.036) | (0.129) |  |
| If dwelling is connected to water system | 0.849 | 0.871 | 0.436 |
|  | (0.030) | (0.035) |  |
| If dwelling is connected to drainage network | 0.298 | 0.321 | 0.721 |
|  | (0.042) | (0.076) |  |
| If cooking fuel of household is electricity or gas | 0.034 | 0.025 | 0.439 |
|  | (0.006) | (0.011) |  |
| Daily per capita expenditure of household (in Quetzales) | 11.841 | 10.177 | 0.043 |
|  | (0.709) | (0.806) |  |
| If household owns TV or radio | 0.741 | 0.742 | 0.966 |
|  | (0.025) | (0.029) |  |
| If household owns a vehicle | 0.240 | 0.223 | 0.622 |
|  | (0.025) | (0.039) |  |
| If household owns livestock | 0.572 | 0.544 | 0.490 |
|  | (0.035) | (0.046) |  |
| Agricultural land size of household (in hectares) | 0.878 | 1.316 | 0.051 |
|  | (0.074) | (0.260) |  |
| If agricultural land has irrigation system | 0.142 | 0.135 | 0.848 |
|  | (0.021) | (0.038) |  |

*(Cont.)*

| **Characteristics at baseline (Nov-Dec 2019)** | **Households** | **Households not** | **p-value** |
| --- | --- | --- | --- |
|  | **included in** | **included in** |  |
|  | **analysis** | **analysis** |  |
| If coffee among one of main crops produced | 0.798 | 0.673 | 0.204 |
|  | (0.043) | (0.124) |  |
| If cardamom among one of main crops produced | 0.116 | 0.289 | 0.122 |
|  | (0.041) | (0.141) |  |
| If corn among one of main crops produced | 0.459 | 0.597 | 0.042 |
|  | (0.038) | (0.079) |  |
| If beans among one of main crops produced | 0.168 | 0.226 | 0.135 |
|  | (0.022) | (0.048) |  |
| If internal migrant in the last 3 years | 0.041 | 0.022 | 0.056 |
|  | (0.006) | (0.008) |  |
| If household is beneficiary of Value Chains Project | 0.442 | 0.314 | 0.072 |
|  | (0.067) | (0.091) |  |
| If received income from agricultural activities | 0.993 | 1.000 | 0.002 |
|  | (0.028) | (0.056) |  |
| If received income from non-agricultural activities | 0.563 | 0.541 | 0.634 |
|  | (0.028) | (0.056) |  |
| If received remittances | 0.253 | 0.211 | 0.254 |
|  | (0.021) | (0.041) |  |
| Observations | 1,824 | 318 |  |

Note: This table reports the results of the orthogonality (balance) test of baseline characteristics between households included and excluded from the analysis. Households excluded from the analysis are households that declined to be surveyed in the follow-up survey, households that did not provide information for all key variables, and mainly households that could not be reached by phone (most often due to permanent low network connectivity in the area). The table reports the corresponding averages and standard errors clustered by community in parentheses. The p-value results from the orthogonality test between the two household groups where a value larger than 0.05 indicates that the average difference in each variable between the two groups is not statistically different at a 95% confidence level.

**Table A.2. Regressions if income decreased on household baseline characteristics**

|  | **(1)** | **(2)** | **(3)** |
| --- | --- | --- | --- |
| **Coefficient** | **If agricultural income decreased** | **If non-agricultural income decreased** | **If remittances decreased** |
| If household head is male | -0.070** | -0.050** | 0.024 |
|  | (0.029) | (0.024) | (0.027) |
| Household head age | -0.001 | -0.001 | -0.001 |
|  | (0.001) | (0.001) | (0.001) |
| If household head did not complete elementary education | 0.036 | 0.058* | -0.023 |
|  | (0.032) | (0.034) | (0.020) |
| If household head completed elementary education or above | 0.029 | 0.079** | -0.003 |
|  | (0.033) | (0.035) | (0.029) |
| If household head main language spoken is Spanish | 0.052 | -0.001 | -0.034 |
|  | (0.051) | (0.052) | (0.047) |
| Household size | 0.000 | 0.005 | 0.004 |
|  | (0.005) | (0.004) | (0.004) |
| If household is beneficiary of social school program | 0.041* | 0.028 | 0.032 |
|  | (0.023) | (0.021) | (0.024) |
| If dwelling has finished walls | 0.014 | -0.001 | 0.017 |
|  | (0.029) | (0.025) | (0.028) |
| If dwelling has finished ceiling | 0.010 | -0.030 | -0.031 |
|  | (0.039) | (0.035) | (0.025) |
| If dwelling has finished floor | 0.026 | -0.006 | 0.025 |
|  | (0.025) | (0.029) | (0.034) |
| If dwelling is connected to electricity | 0.113*** | 0.077** | 0.049 |
|  | (0.029) | (0.031) | (0.059) |
| If dwelling is connected to water system | 0.000 | 0.013 | -0.020 |
|  | (0.033) | (0.039) | (0.042) |
| If dwelling is connected to drainage network | 0.015 | 0.028 | 0.019 |
|  | (0.031) | (0.028) | (0.030) |
| If cooking fuel of household is electricity or gas | -0.004 | 0.077 | -0.123 |
|  | (0.057) | (0.047) | (0.088) |
| If Tercile 2 Daily per capita expenditure | -0.072*** | -0.007 | -0.025 |
|  | (0.025) | (0.026) | (0.037) |
| If Tercile 3 Daily per capita expenditure | -0.018 | 0.025 | -0.007 |
|  | (0.031) | (0.030) | (0.029) |
| If household owns TV or radio | 0.004 | -0.017 | 0.022 |
|  | (0.024) | (0.022) | (0.045) |
| If household owns a vehicle | 0.046 | 0.016 | 0.019 |
|  | (0.029) | (0.032) | (0.024) |
| If household owns livestock | 0.021 | 0.041* | -0.002 |
|  | (0.027) | (0.023) | (0.027) |

*(Cont.)*

|  | **(1)** | **(2)** | **(3)** |
| --- | --- | --- | --- |
| **Coefficient** | **If agricultural income decreased** | **If non-agricultural income decreased** | **If remittances decreased** |
| Ln agricultural land size of household (in hectares) | -0.016 | -0.007 | -0.001 |
|  | (0.011) | (0.013) | (0.011) |
| If agricultural land has irrigation system | 0.036 | 0.015 | 0.022 |
|  | (0.036) | (0.032) | (0.032) |
| If coffee among one of main crops produced | 0.027 | -0.007 | -0.066** |
|  | (0.044) | (0.033) | (0.030) |
| If cardamom among one of main crops produced | -0.007 | 0.097* | 0.100 |
|  | (0.026) | (0.050) | (0.071) |
| If corn among one of main crops produced | 0.020 | 0.001 | -0.006 |
|  | (0.023) | (0.024) | (0.019) |
| If beans among one of main crops produced | 0.010 | -0.000 | 0.013 |
|  | (0.027) | (0.023) | (0.021) |
| If internal migrant in household last 3 years | 0.112** | 0.132** | 0.056 |
|  | (0.048) | (0.054) | (0.051) |
| If household is beneficiary of Value Chains Project | 0.040 | 0.271*** | -0.138*** |
|  | (0.041) | (0.043) | (0.048) |
| Constant | 0.461*** | 0.251*** | 0.951*** |
|  | (0.082) | (0.094) | (0.085) |
| Community Fixed Effects | Yes | Yes | Yes |
| Observations | 1,824 | 1,824 | 578 |
| R-squared | 0.335 | 0.292 | 0.349 |

Note: This table reports the regression results of estimating by ordinary least squares if the income source decreased after the lockdown on a set of household characteristics at baseline. Robust standard errors reported in parentheses clustered by community. *** p<0.01, ** p<0.05, * p<0.1. The dependent variable is equal to one if the household reported a large or small decrease in each corresponding income source, and zero otherwise.

**Table A.3. Regressions if dietary diversity decreased on household baseline characteristics**

|  | **(1)** | **(2)** | **(3)** | **(4)** | **(5)** |
| --- | --- | --- | --- | --- | --- |
| **Coefficient** | **If HDDS decreased** | **If ASF score decreased** | **If F&V score decreased** | **If WDDS decreased** | **If children DDS decreased** |
| If household head is male | 0.024 | -0.011 | -0.050 | -0.017 | -0.110 |
|  | (0.036) | (0.031) | (0.034) | (0.034) | (0.082) |
| Household head age | -0.000 | -0.001 | -0.001 | -0.001 | -0.005* |
|  | (0.001) | (0.001) | (0.001) | (0.001) | (0.003) |
| If household head did not complete elementary education | -0.041 | 0.002 | -0.044 | -0.025 | -0.056 |
|  | (0.028) | (0.028) | (0.029) | (0.028) | (0.099) |
| If household head completed elementary education or above | -0.050 | 0.044 | -0.030 | -0.026 | -0.193** |
|  | (0.034) | (0.029) | (0.033) | (0.036) | (0.092) |
| If household head main language spoken is Spanish | -0.018 | -0.032 | 0.047 | 0.045 | 0.075 |
|  | (0.064) | (0.068) | (0.049) | (0.051) | (0.119) |
| Household size | 0.007 | 0.012** | 0.001 | 0.012** | 0.017 |
|  | (0.006) | (0.006) | (0.006) | (0.005) | (0.013) |
| If household is beneficiary of social school program | 0.003 | -0.052* | -0.032 | -0.023 | -0.095 |
|  | (0.028) | (0.029) | (0.028) | (0.029) | (0.078) |
| If dwelling has finished walls | -0.047 | -0.031 | -0.014 | -0.051 | 0.017 |
|  | (0.031) | (0.027) | (0.031) | (0.035) | (0.071) |
| If dwelling has finished ceiling | -0.005 | -0.004 | 0.014 | 0.029 | -0.154 |
|  | (0.040) | (0.038) | (0.040) | (0.051) | (0.111) |
| If dwelling has finished floor | 0.056 | 0.064** | -0.012 | 0.049* | -0.093 |
|  | (0.036) | (0.028) | (0.029) | (0.028) | (0.088) |
| If dwelling is connected to electricity | 0.105* | 0.048 | -0.043 | 0.067 | 0.084 |
|  | (0.053) | (0.050) | (0.057) | (0.061) | (0.098) |
| If dwelling is connected to water system | 0.065 | -0.012 | 0.038 | 0.035 | 0.091 |
|  | (0.049) | (0.048) | (0.039) | (0.043) | (0.099) |
| If dwelling is connected to drainage network | -0.117*** | -0.060* | -0.055* | -0.076** | -0.041 |
|  | (0.037) | (0.034) | (0.028) | (0.033) | (0.106) |
| If cooking fuel of household is electricity or gas | 0.064 | 0.019 | 0.048 | 0.075 | 0.205* |
|  | (0.062) | (0.066) | (0.066) | (0.060) | (0.116) |
| If Tercile 2 Daily per capita expenditures | 0.066* | 0.082** | 0.040 | 0.071* | 0.085 |
|  | (0.038) | (0.037) | (0.031) | (0.038) | (0.074) |
| If Tercile 3 Daily per capita expenditures | 0.107** | 0.166*** | 0.078** | 0.153*** | 0.062 |
|  | (0.042) | (0.040) | (0.033) | (0.044) | (0.083) |
| If household owns TV or radio | 0.010 | 0.002 | -0.022 | 0.029 | 0.023 |
|  | (0.025) | (0.027) | (0.028) | (0.026) | (0.078) |
| If household owns a vehicle | 0.042 | 0.016 | 0.001 | 0.035 | 0.047 |
|  | (0.030) | (0.036) | (0.030) | (0.034) | (0.082) |
| If household owns livestock | 0.029 | 0.018 | 0.019 | 0.034 | 0.009 |
|  | (0.033) | (0.028) | (0.030) | (0.030) | (0.071) |

*(Cont.)*

|  | **(1)** | **(2)** | **(3)** | **(4)** | **(5)** |
| --- | --- | --- | --- | --- | --- |
| **Coefficient** | **If HDDS decreased** | **If ASF score decreased** | **If F&V score decreased** | **If WDDS decreased** | **If children DDS decreased** |
| Ln agricultural land size of household (in hectares) | 0.006 | 0.010 | 0.016 | 0.002 | 0.022 |
|  | (0.013) | (0.016) | (0.015) | (0.016) | (0.020) |
| If agricultural land has irrigation system | 0.004 | 0.020 | 0.037 | 0.016 | 0.066 |
|  | (0.031) | (0.035) | (0.037) | (0.042) | (0.099) |
| If coffee among one of main crops produced | 0.005 | -0.014 | 0.054 | 0.058 | 0.078 |
|  | (0.049) | (0.045) | (0.047) | (0.062) | (0.135) |
| If cardamom among one of main crops produced | 0.004 | 0.057 | -0.054 | -0.034 | -0.190* |
|  | (0.076) | (0.071) | (0.043) | (0.054) | (0.101) |
| If corn among one of main crops produced | -0.056** | -0.052* | -0.033 | -0.028 | -0.048 |
|  | (0.026) | (0.027) | (0.043) | (0.035) | (0.073) |
| If beans among one of main crops produced | 0.026 | -0.020 | 0.004 | 0.012 | -0.058 |
|  | (0.034) | (0.033) | (0.036) | (0.038) | (0.094) |
| If internal migrant in household last 3 years | 0.034 | 0.004 | 0.074 | 0.007 | 0.118 |
|  | (0.060) | (0.064) | (0.071) | (0.065) | (0.220) |
| If household is beneficiary of Value Chains Project | -0.335*** | -0.276*** | 0.154*** | -0.016 | -0.011 |
|  | (0.050) | (0.052) | (0.052) | (0.070) | (0.257) |
| Constant | 0.436*** | 0.638*** | 0.348*** | 0.287** | 1.105*** |
|  | (0.101) | (0.094) | (0.102) | (0.126) | (0.290) |
| Community Fixed Effects | Yes | Yes | Yes | Yes | Yes |
| Observations | 1,824 | 1,824 | 1,824 | 1,603 | 318 |
| R-squared | 0.176 | 0.172 | 0.110 | 0.151 | 0.397 |

Note: This table reports the regression results of estimating by ordinary least squares if the dietary diversity score decreased after the lockdown on a set of household characteristics at baseline. Robust standard errors reported in parentheses clustered by community. *** p<0.01, ** p<0.05, * p<0.1. The dependent variable is equal to one if the household shows a decrease in each corresponding dietary diversity score, and zero otherwise. The HDDS, ASF, and F&V scores measure the number of food groups that a member of the household consumed over the previous 24 hours, while the WDDS and children DDS measure the number of food groups that a selected woman 15-49 years old or a child 6-23 months old in the household consumed over the previous 24 hours. See the main text for the groups comprised in each of the scores.
